# Quantitative study and related factors analysis of sciatic neuropathy in type 2 diabetes mellitus patients by elastic imaging virtual tissue imaging quantification technique

**DOI:** 10.1186/s41065-025-00565-7

**Published:** 2025-10-10

**Authors:** Ruixing Liu, Beibei Yu, Gaopan Cao, Lina Ye, Dan Zhang

**Affiliations:** 1 Departments of Ultrasonography, The Second Affiliated Hospital of Wenzhou Medical University, No.109, West Xueyuan Road, Wenzhou, 325027 Zhejiang China; 2 Wenzhou Dongtou People’s Hospital, Wenzhou, 325700 Zhejiang China

**Keywords:** Diabetes mellitus, Peripheral nervous system diseases, Sciatic neuropathy, Ultrasonography, Elastography, Shear wave velocity, Virtual tissue imaging quantification

## Abstract

**Background:**

The incidence of diabetic peripheral neuropathy (DPN) is increasing every year for type 2 diabetes mellitus (T2DM) patients, and diabetic polyneuropathy is a common type.

**Objective:**

To quantify and analyze the factors associated with diabetic polyneuropathy using the virtual tissue imaging quantification (VTIQ) technique.

**Method:**

182 patients with T2DM, 137 patients with diabetic polyneuropathy, and 198 healthy volunteers were included in this retrospective cross-sectional diagnostic study. Sciatic neuropathy was evaluated through Doppler ultrasound examination with a VTIQ quantitative analysis system to acquire elastic modulus, cross-sectional area (CSA) and shear wave velocity (SWV). Nerve conduction velocity (NCV) was also evaluated via neurophysiological examination. Logistic regression was used to analyze odds ratios (OR) related diabetic polyneuropathy. The diagnostic accuracy of the VTIQ technique-acquired index on diabetic polyneuropathy was analyzed using the receiver operating characteristic (ROC) curve.

**Results:**

VTIQ technique-acquired indexes all differed significantly among three study groups, among which Elastic modulus and CSA were independently related to diabetic polyneuropathy risk according to the logistic regression analysis. NCV was also an independent risk factor for diabetic polyneuropathy. ROC analysis revealed that Elastic modulus, CSA and NCV can distinguish diabetic polyneuropathy patients from T2DM cases with the AUC of 0.797, 0.654 and 0.775 respectively. But their combination achieved the highest diagnostic value (AUC = 0.883). CSA and SWV of the sciatic nerve are positively correlated with visual analog scale (VAS) scores.

**Conclusion:**

VTIQ technology contributes to the diagnosis of diabetic polyneuropathy, it can improve the diagnostic value of neurophysiological examination on sciatic neuropathy for T2DM patients.

**Supplementary Information:**

The online version contains supplementary material available at 10.1186/s41065-025-00565-7.

## Introduction

Diabetic peripheral neuropathy (DPN) is one of the most common complications of diabetes mellitus (DM), and its incidence gradually increases with the prolongation of DM [1–5]. The rate of DPN in DM has been reported to range from 16 to 66% abroad, while the rate in China ranges from 10.31 to 69.2% [6–11]. Diabetic polyneuropathy is a common type of DPN, mainly due to long-term high blood sugar caused by nerve damage. In diabetic patients, the incidence of sciatic neuropathy is high, which seriously affects the quality of life of patient [12]. The clinical manifestations of DPN patients are lower extremity pain, paresthesia, motor disorders, and so on [13]. Its pathogenesis includes neurometabolic disorders and microcirculatory disorders caused by hyperglycemia [14]. In addition, autoimmune factors and genetic factors also play a role in the development of DPN. Patients should actively control blood sugar, take nutritional nerve, improve microcirculation, pain treatment and other comprehensive treatment measures, and pay attention to prevent complications.

At present, clinical diagnosis of peripheral neuropathy is based on history, clinical symptoms, nerve biopsy, magnetic resonance imaging (MRI), high-frequency ultrasound (HFUS), and neuro-electrophysiological examination (NEE) [15, 16]. The NEE is the gold standard for the diagnosis of peripheral neuropathy, which can reflect the function of the nerves but cannot show the morphological and structural changes of the nerves. It can be easily affected by anatomical variations, the external environment, and the patient’s conditions, with a high false-negative rate [17]. MRI can visualize changes in the macrostructure and microstructure of the nerves, which helps in the diagnosis, characterization, and localization of peripheral nerve diseases, but it is difficult to quantify, in some circumstances it may have limited specificity, and is not widely used in the clinic because of its high cost, long time and many contraindications [15]. HFUS can visually and clearly show the continuity of small nerves, the echogenicity inside the nerves, the relationship between the outer membrane of the nerves and the adjoining tissues, and the location of the lesion, and judge the patient’s neuropathy by comparing the nerves bilaterally. In the context of ultrasound imaging, classical signs of diabetic neuropathy include fascicular enlargement, increased cross-sectional area (CSA), and loss of the normal “honeycomb” echotexture. These hallmark patterns were first documented over a decade ago [18] and have been validated in multiple follow-up studies [19–21]. It has the advantages of simplicity, low cost, and non-invasiveness compared with other examinations, but it cannot react to the stiffness of the nerves [16, 17, 22–26]. Nerve biopsy is the gold standard for the diagnosis of small nerve fiber lesions and is highly reliable and accurate in assessing the extent of damage to the nerve tissue sampled from the patient, but only samples a specific nerve node and only detects pathological changes in the nerve fibers of the sampled segment and does not reflect the full extent of the damage to the nerve [27, 28].

Virtual tissue imaging quantification (VTIQ) is an ultrasound elastography technique that evaluates the hardness of tissues by measuring shear wave velocity (SWV). In medical research, the technique is applied to assess tissue changes in various disease states. In patients with carpal tunnel syndrome (CTS), the nerve SWV of patients with CTS is higher than control group, indicating the reproducibility of VTIQ diagnostic technique [29]. In addition, another study also reports that combination of VTIQ can improve the diagnostic efficiency of HFUS for CTS [30]. Therefore, the application value of VTIQ technique in DPN attracts our interest. In the current study, T2DM patients with or without diabetic polyneuropathy were enrolled, and received VTIQ analysis to detect sciatic neuropathy. Then the diagnostic value of VTIQ-acquired indexes was evaluated, and compared with neurophysiological examination.

## Materials and methods

### Study design

This study was designed as a retrospective cross-sectional diagnostic study. A total of 182 T2DM patients without any complication (T2DM group), 137 patients with diabetic polyneuropathy (DPN group), and 198 healthy volunteers (healthy group) of the same period from The Second Affiliated Hospital of Wenzhou Medical University were randomly collected as study subjects. All participants signed an informed consent form. This retrospective study was approved by the Ethics Committee of The Second Affiliated Hospital of Wenzhou Medical University. Participants excluded at each stage, across the groups were provided in Supplementary Fig. 1.

### Inclusion and exclusion criteria

Inclusion and exclusion criteria for healthy controls were as follows. Inclusion criteria: [1] Age over 20 years; [2] No history of metabolic diseases such as diabetes, cardiovascular disease, chronic kidney diseases; [3] Body mass index (BMI) between 18.5 and 24.9. Exclusion criteria: [1] Presence of acute or chronic diseases affecting participants health; [2] Recent use of medications affecting metabolism; [3] Pregnant or breastfeeding females; and [4] Patients involved in other clinical trials.

Inclusion and exclusion criteria for the T2DM group were as follows. Inclusion criteria: [1] Confirmed diagnosis of T2DM, meeting the 2019 American Diabetes Association (ADA) criteria (fasting blood glucose ≥ 7.0 mmol/L, random blood glucose ≥ 11.1 mmol/L, or glycated hemoglobin (HbA1c) ≥ 6.5%); [2] Aged 20 years or older; and [3] current use of stable glucose-lowering medication for at least 3 months. Exclusion criteria: [1] Presence of serious complications, such as diabetic nephropathy, diabetic retinopathy; [2] Having other diseases that might affect the nerve functions; [3] Have serious systemic diseases, such as heart failure, liver and kidney dysfunction, and severe cardiovascular disease; [4] Has a history of alcohol abuse or drug addiction; [5] Have neurological disorders, such as stroke, Parkinson’s disease; [6] Have a history of mental illness; [7] Have malignant tumors; [8] Have immune system diseases or are undergoing immunosuppressive treatment; [9] Have used drugs that may affect nerve function recently (within 3 months) [10]. obtained major surgery or hospitalization in the last 6 months; [11] Comorbidities with other endocrine disorders; and [12] Pregnant or breastfeeding women.

Inclusion and exclusion criteria for the DPN group were as follows. Inclusion criteria: [1] Meet the inclusion criteria of the T2DM group, meeting the 2019 ADA criteria; [2] Neuropathy at or after the diagnosis of DM; [3] Patients were diagnosed by NEE or B-mode ultrasonography; [4] Clinical symptoms of neuropathy occur (such as pain, numbness, and sensory abnormalities) combined with any one abnormality of the five examinations including ankle reflex (Taylor Percussion Reflex-Hammer), pinprick nociception detected by 28-gauge dedicated needle, 128 Hz tuning fork for vibration sensation, pressure sensation using 10-g monofilament, and temperature sensation detected by thermal aesthesiometer or cold/hot metal rods on the big toes; and [5] if there are no clinical symptoms, at least two abnormal results among the five tests can also lead to diagnosis. Exclusion criteria: [1] Have a history of neurological disorders or abnormal manifestations of neurological functions; [2] consistent with the exclusion criteria of T2DM group.

### Data collection

The basic information of the participant such as height, weight, and sex was collected. Triglycerides (TG), high-density lipoprotein cholesterol (HDL-C), low-density lipoprotein cholesterol (LDL-C), Superoxide dismutase (SOD), glutathione antioxidant enzyme (GSH-Px), catalase (CAT), and other biochemicals were measured in peripheral venous blood of participants.

### Ultrasound and neurophysiological examination of sciatic nerve

**Fig. 1:**
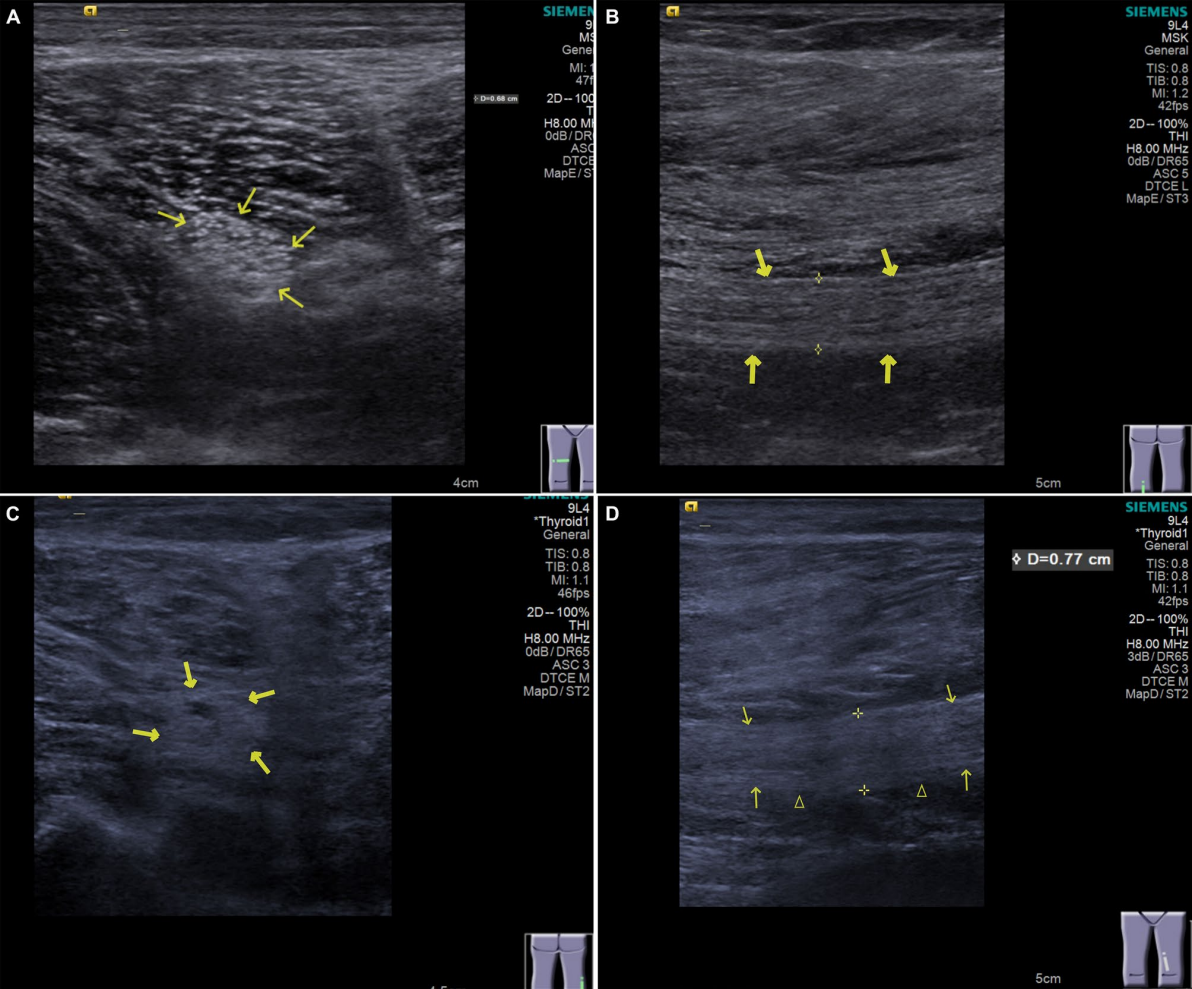
Grey-scale ultrasound images of DPN and T2DM patients. (**A**) The internal echoes of cross-sectional B-ultrasound images of T2DM show a regular honeycomb pattern (presented by arrows). (**B**) The longitudinal section image of T2DM patients (presented by arrows). (**C**) The internal echoes in the cross-sectional images of DPN patients show liquefaction (presented by arrows). (**D**) The longitudinal section image of DPN patients. The nerve bundle between the triangles is enlarged and blurred texture, arrows indicate the width and clear texture of normal nerve bundle

The grey-scale ultrasound images for classical signs of DPN patients, including fascicular enlargement, increased CSA, and loss of the normal “honeycomb” echotexture have been carefully evaluated. These features were annotated on the images to ensure clear visualization and accurate interpretation (Fig. 1). The sonographers were specifically trained to identify and document these features, and the images were reviewed by a senior radiologist to ensure consistency and accuracy.

All ultrasonic examinations were performed by two professional sonographers each with more than 5 years of experience in musculoskeletal nerve ultrasound. The sonographers were blinded to the clinical status of the subjects. A Siemens OXANA2 color Doppler ultrasound machine was used, with an L12-5 line-array probe at a frequency of 5–12 MHz and a VTIQ quantitative analysis system. To ensure consistency in image acquisition and analysis across all subjects, a detailed elastographic imaging protocol was established. Pressure standardization was achieved by applying a consistent pressure with the probe, ensuring stable and clear elastographic images. The region of interest (ROI) was selected in the mid-thigh cross-section of the sciatic nerve, avoiding blood vessels and fat tissue. A circular ROI with a diameter of 1 cm was used, and its size and position were kept consistent across all subjects. To guarantee the reproducibility of the data, triple measurements were taken from the same area in each subject, with the mean value used as the final result.

**Fig. 2:**
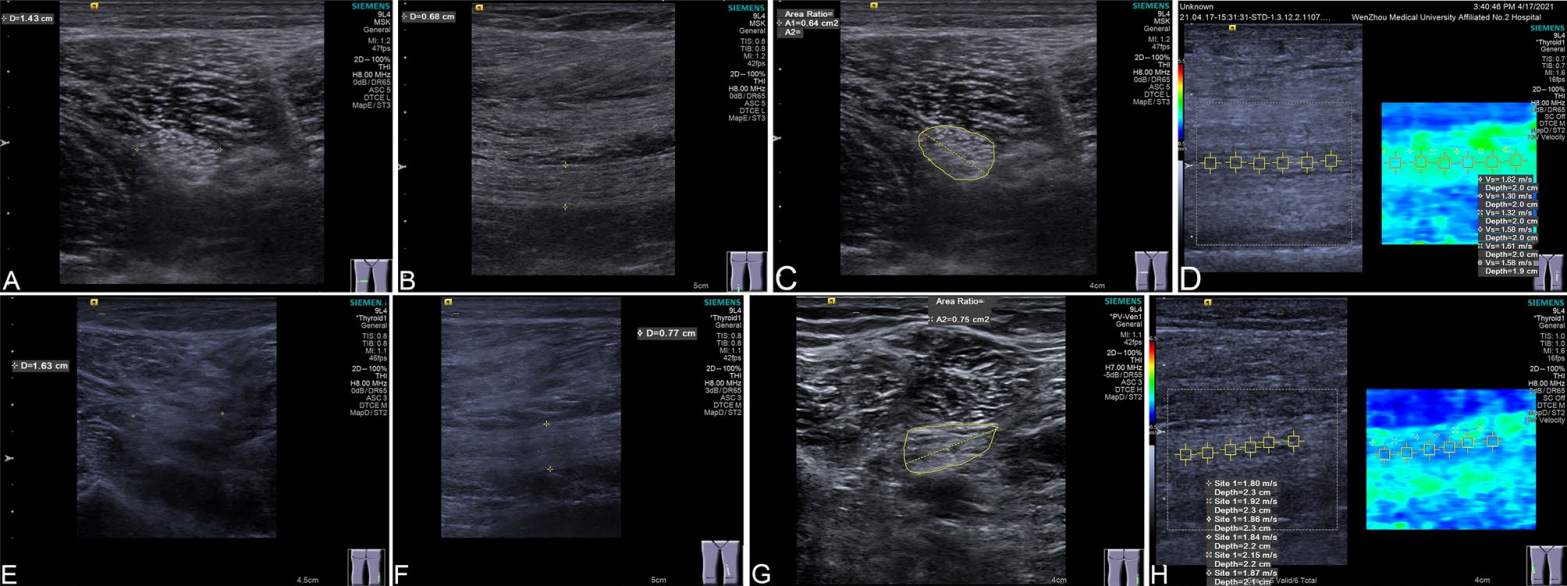
Representative figures of color Doppler ultrasound with a VTIQ quantitative analysis system. The cross-sectional area (CSA) of sciatic nerve was measured by Trace method. The shear wave velocity (SWV) was detected by VTIQ technique. (**A**) Width of sciatic nerve in T2DM patients without DPN. The largest diameter is 1.43 cm and presented by yellow crossed-stripe symbol. (**B**) Thickness of sciatic nerve in T2DM patients without DPN. The largest diameter is 0.68 cm and presented by yellow crossed-stripe symbol. (**C**) CAS of T2DM patient was marked by a yellow circle (the area = 0.64 m^2^). The dotted line indicated the largest diameter. (**D**) SWV of T2DM patient was ranged from 1.30 to1.62 m/s. (**E**) Width of sciatic nerve in DPN patient. The largest diameter is 1.63 cm and presented by yellow crossed-stripe symbol. (**F**) Thickness of sciatic nerve in DPN patient. The largest diameter is 0.77 cm and presented by yellow crossed-stripe symbol. (**G**) CAS of DPN patient was marked by a yellow circle (the area = 0.75 m^2^). The dotted line indicated the largest diameter. (**H**) SWV of DPN patient was ranged from 1.80 to 2.15 m/s

The first high-frequency ultrasound examination was performed by selecting a clear image of the sciatic nerve in the mid-thigh cross-section for freezing, and measuring the anteroposterior and posterior diameters of the right and left sciatic nerves, the transverse diameters of the right and left sciatic nerves, and the cross-cutting area of the right and left sciatic nerves. In the same position as the high-frequency ultrasound examination, the long axis section of the sciatic nerve was obtained at the marking place on the body surface. The cross-sectional area (CSA) of sciatic nerve was measured by Trace method. Each measurement was repeated 7 times, the average value was taken, and the measurement location was marked on the body surface. The long axis section of sciatic nerve was obtained at the marks on the body surface in the same position as high-frequency ultrasound examination. Then the VTIQ technique was activated to keep the sound beam perpendicular and fixed to the swept area, and the shear wave velocity (SWV) was detected. The average value of the same area was measured at least six times as the outcome, and the mean value of each measurement was taken. Representative figures of color Doppler ultrasound with a VTIQ quantitative analysis system were shown in Fig. 2. Neurophysiological examination was used to detect nerve conduction velocity (NCV).

### Data analysis

SPSS 22.0 statistical software was applied for data analysis. Measurement information was expressed as mean ± standard deviation (SD). Continuous variables data were analyzed by one-way analysis of variance (ANOVA) or non-parametric test for differences among differences among 3 groups or above. Differences in qualitative variables were analyzed by chi-square test (χ^2^), and risk factors were explored by logistic regression (enter method for univariate analysis, forward stepwise likelihood ratio method for multivariate analysis) to analyze the correlation between each clinical characteristic and diabetic polyneuropathy in terms of odds ratio (OR) and 95% confidence interval (CI). The receiver operating characteristic (ROC) curve was used to analyze the predictive effect of each indicator on diabetic polyneuropathy. Pearson correlation analysis assessed the correlation between the VTIQ measures and the visual analog scale (VAS) scores. *P* < 0.05 was considered statistically significant.

## Result

### Comparison of general indicators

Statistical power was calculated by GPower 3.1, using F tests. When effect size = 0.25 and α = 0.05, the statistical power was 0.9996. Baseline and clinical characteristics were compared among three groups. Age (*P* = 0.510), gender (*P* = 0.883), BMI(*P* = 0.815), hypertension (*P* = 0.965), and hyperlipidemia (*P* = 0.541) were not statistically significant among the three groups. T2DM duration (*P* < 0.001), fasting plasma glucose (FPG, *P* < 0.001), 2 h postprandial plasma glucose (2hPPG, *P* < 0.001), HbA1c (*P* < 0.001), TG (*P* = 0.005), LDL-C (*P* < 0.001) were significantly related with diabetic polyneuropathy. Oxidative stress and inflammatory factors were found to be highly correlated with diabetic polyneuropathy (Table 1). Thickness, width, CSA, and SWV of both sides of the sciatic nerve are detected using VTIQ (Fig. 1) and they were significantly higher in DPN patients than other two groups (Table 1). Thickness, width, CSA, and SWV measurements on both sides of the sciatic nerve were relatively small in the healthy control group and increased only in the T2DM and DPN group, whereas the increase was greater in the group of patients (Supplementary Table 1).

**Table 1:** Baseline and clinical characteristics of diabetic polyneuropathy patients

Characteristics	Control (*n* = 198)	T2DM (*n* = 182)	DPN (*n* = 137)	*P* ^a^	*P* ^b^	*P* ^c^
Age	55.63 ± 10.01	54.66 ± 10.54	54.47 ± 9.33	0.362	0.288	0.867
> 60	68(34.34)	56(30.77)	44(32.12)			
≤ 60	130(65.66)	126(69.23)	93(67.88)			
Gender				0.887	0.623	0.723
Male	103(52.02)	96(52.75)	75(54.74)			
Female	95(47.98)	86(47.25)	62(45.26)			
BMI (kg/m^2^)	23.41 ± 2.80	23.48 ± 2.89	23.27 ± 3.02	0.938	0.553	0.597
T2DM duration (months)	-	68.27 ± 31.34	86.34 ± 32.54	/	/	< 0.001
> 120	-	8(4.40)	18(13.14)			
≤ 120	-	174(95.60)	119(86.86)			
Hypertension				0.980	0.820	0.806
Yes	47(23.74)	43(23.63)	34(24.82)			
No	151(76.26)	139(76.37)	103(75.18)			
Hyperlipidemia				0.364	0.329	0.894
Yes	79(39.90)	81(44.51)	62(45.26)			
No	119(60.10)	101(55.49)	75(54.74)			
FPG (mmol/L)	4.4–7.8	8.46 ± 2.36	8.52 ± 2.71	< 0.001	< 0.001	0.592
2hPPG (mmol/L)	5.40 ± 1.82	8.34 ± 2.28	8.79 ± 2.15	< 0.001	< 0.001	0.074
> 10.0	0	43(23.63)	40(29.20)			
≤ 10.0	198	139	97			
HbA1c	5.40 ± 1.82	8.34 ± 2.28	8.79 ± 2.15	< 0.001	< 0.001	0.626
TG (mmol/L)	1.28 ± 0.49	1.31 ± 0.55	1.46 ± 0.55	0.492	0.001	0.011
LDLC (mmol/L)	2.51 ± 0.96	3.87 ± 0.98	4.15 ± 1.06	< 0.001	< 0.001	0.018
**Oxidative stress**						
SOD (U/mL)	180.24 ± 36.42	157.26 ± 36.62	99.79 ± 30.56	< 0.001	< 0.001	< 0.001
GSH-Px(U/L)	511.76 ± 158.35	468.52 ± 109.73	391.14 ± 93.15	0.004	< 0.001	< 0.001
CAT (U/L)	33.79 ± 14.64	75.60 ± 15.74	57.64 ± 12.83	< 0.001	< 0.001	< 0.001
**Inflammatory factors**						
CRP (mg/L)	1.87 ± 0.67	6.54 ± 2.46	10.66 ± 3.70	< 0.001	< 0.001	< 0.001
TNF-α (ng/L)	4.27 ± 2.55	11.05 ± 3.95	16.34 ± 4.39	< 0.001	< 0.001	< 0.001
IL-6 (ng/L)	2.47 ± 1.00	7.66 ± 2.48	10.31 ± 2.64	< 0.001	< 0.001	< 0.001
**VTIQ index**						
Width (mm)						
Left	13.25 ± 1.26	13.99 ± 2.01	14.70 ± 2.10	< 0.001	< 0.001	0.002
Right	13.42 ± 1.28	13.96 ± 1.98	14.81 ± 1.99	0.002	< 0.001	< 0.001
Thickness (mm)						
Left	5.16 ± 0.62	5.76 ± 0.87	6.86 ± 1.19	< 0.001	< 0.001	< 0.001
Right	5.20 ± 0.68	5.92 ± 0.94	7.02 ± 1.25	< 0.001	< 0.001	< 0.001
CSA (mm^2^)						
Left	54.67 ± 13.63	64.03 ± 14.19	70.61 ± 13.42	< 0.001	< 0.001	< 0.001
Right	55.54 ± 12.99	64.36 ± 13.36	72.00 ± 14.78	< 0.001	< 0.001	< 0.001
SWV (m/s)						
Left	1.25 ± 0.21	1.55 ± 0.24	1.80 ± 0.23	< 0.001	< 0.001	< 0.001
Right	1.27 ± 0.22	1.55 ± 0.24	1.83 ± 0.25	< 0.001	< 0.001	< 0.001
NCV (m/s)						
Left	49.87 ± 8.40	45.17 ± 6.48	38.01 ± 6.08	< 0.001	< 0.001	< 0.001
Right	50.20 ± 9.31	45.59 ± 6.49	38.72 ± 6.56	< 0.001	< 0.001	< 0.001
VAS score	-	-	4.77 ± 1.98			

**Notes**: T2DM, type 2 diabetes mellitus; DPN, diabetic polyneuropathy; BMI, body mass index; FPG, fasting plasma glucose; 2hPPG, 2 h postprandial plasma glucose; HbA1c, glycosylated hemoglobin; TG, triglyceride; LDLC, low-density lipoprotein cholesterol; HCY, homocysteine; SOD, superoxide dismutase; GSH-Px, glutathione peroxidase; CAT, catalase; CRP, C-reactive protein; TNF-α, tumor necrosis factor-α; IL-6, interleukin-6; VTIQ, virtual touch tissue imaging quantification; CSA, cross-sectional area; SWV, shear wave velocity; NCV, nerve conduction velocity; VAS, visual analog scale; a, T2DM vs. control; b, DPN vs. control; c, DPN vs. T2DM

### Ultrasound and neurophysiological examination of sciatic nerve

As shown in the grey-scale B mode ultrasound, regular honeycomb pattern (Fig. 1A) was presented in the cross-section of sciatic nerve in T2DM patients who without DPN. Meanwhile, the longitudinal section image showed that the nerve fibers are continuous and the echoes are uniform (Fig. 1B). In contrast, DPN patients showed liquefaction in the internal echoes, indicating a loss of the normal honeycomb pattern (Fig. 1C). The nerve fascicles were enlarged and had blurred texture in the longitudinal section (Fig. 1D).

The VTIQ quantitative analysis system further quantified these differences. In T2DM patients, the width of the sciatic nerve was 1.43 cm (Fig. 2A), the thickness was 0.68 cm (Fig. 2B), the CSA was 0.64 cm² (marked by a yellow circle, Fig. 2C), and the SWV ranged from 1.30 to 1.62 m/s (Fig. 2D). In contrast, DPN patients exhibited increased nerve width (Fig. 2E, 1.63 cm), thickness (Fig. 2F, 0.77 cm), and CSA (0.75 cm², marked by a yellow circle, Fig. 2G), with SWV values ranging from 1.80 to 2.15 m/s (Fig. 2H). These findings indicate increased stiffness and reduced elasticity of the sciatic nerve in DPN patients.

### Analysis of clinical characteristics and diabetic polyneuropathy

Univariate analysis showed that T2DM duration (*P* = 0.007), TG (*P* = 0.016), SOD (*P* = 0.034), GSH-Px (*P* = 0.024), CAT (*P* = 0.031), C-reactive protein (CRP, *P* = 0.028), tumor necrosis factor-α (TNF-α, *P* = 0.024), CSA (*P* < 0.05), SWV (*P* = 0.011), NCV (*P* < 0.05) were risk factors for diabetic polyneuropathy development (Table 2). T2DM duration (OR: 3.290, 95% CI, 1.385–7.812), TG (OR: 1.738, 95% CI, 1.111–2.719), CRP (OR: 1.648, 95% CI, 1.054–2.578), TNF-α (OR: 1.676, 95% CI, 1.071–2.621), left sciatic nerve CSA (OR: 1.698, 95% CI, 1.086–2.657), right sciatic nerve CSA ( OR: 1.929, 95% CI, 1.230–3.024), and SWV of the right sciatic nerve (OR: 1.793, 95% CI, 1.144–2.812) were positively correlated with the odds of diabetic polyneuropathy development. But SOD (OR: 0.609, 95% CI, 0.386–0.963), GSH-Px (OR: 0.596, 95% CI, 0.380–0.934), CAT (OR: 0.611, 95% CI, 0.391–0.955), left sciatic NCV (OR: 0.617, 95% CI, 0.394–0.968), and right sciatic NCV (OR: 0.522, 95% CI, 0.333–0.818) were negatively linked with the risk of diabetic polyneuropathy.

**Table 2:** Logistic regression analysis for the association of clinic features with diabetic polyneuropathy risk

Characteristics	Univariate analysis		Multivariate analysis	
	OR (95%CI)	*P*	OR (95%CI)	*P*
Age (> 60)	1.065(0.661–1.715)	0.797		
Gender (female)	0.923(0.591–1.440)	0.723		
BMI (high)	1.153(0.737–1.803)	0.533		
T2DM duration (> 120)	3.290(1.385–7.812)	0.007	3.285(1.276–8.453)	0.014
Hypertension (yes)	1.037(0.666–1.616)	0.872		
Hyperlipidemia (yes)	1.333(0.806–2.204)	0.262		
FPG (high)	1.037(0.666–1.616)	0.872		
2hPPG (> 10.0)	0.827(0.530–1.289)	0.401		
TG (high)	1.738(1.111–2.719)	0.016	1.998(1.224–3.262)	0.006
LDLC (high)	1.246(0.799–1.942)	0.333		
SOD (high)	0.609(0.386–0.963)	0.034	0.592(0.359–0.975)	0.039
GSH-Px (high)	0.596(0.380–0.934)	0.024	0.555(0.337–0.913)	0.020
CAT (high)	0.611(0.391–0.955)	0.031	/	/
CRP (high)	1.648(1.054–2.578)	0.028	1.641(1.007–2.673)	0.047
TNF-α (high)	1.676(1.071–2.621)	0.024	1.638(1.005–2.669)	0.048
IL-6 (high)	1.015(0.651–1.582)	0.947		
Width (mm)				
Left (high)	1.465(0.938–2.287)	0.093		
Right (high)	1.219(0.782-1.900)	0.382		
Thickness (mm)				
Left	1.311(0.841–2.045)	0.232		
Right	1.030(0.661–1.606)	0.895		
CSA (mm^2^)				
Left	1.698(1.086–2.657)	0.020	/	/
Right	1.929(1.230–3.024)	0.004	1.892(1.161–3.082)	0.010
SWV (m/s)				
Left	1.274(0.817–1.987)	0.285		
Right	1.793(1.144–2.812)	0.011	1.898(1.160–3.107)	0.011
NCV (m/s)				
Left	0.617(0.394–0.968)	0.036	/	/
Right	0.522(0.333–0.818)	0.005	0.522(0.320–0.853)	0.009

**Notes**: T2DM, type 2 diabetes mellitus; DPN, diabetic polyneuropathy; BMI, body mass index; FPG, fasting plasma glucose; 2hPPG, 2 h postprandial plasma glucose; HbA1c, glycosylated hemoglobin; TG, triglyceride; LDLC, low-density lipoprotein cholesterol; HCY, homocysteine; SOD, superoxide dismutase; GSH-Px, glutathione peroxidase; CAT, catalase; CRP, C-reactive protein; TNF-α, tumor necrosis factor-α; IL-6, interleukin-6; VTIQ, virtual touch tissue imaging quantification; CSA, cross-sectional area; SWV, shear wave velocity; NCV, nerve conduction velocity; OR, odds ratio; 95%CI, 95% confidence interval; /, excluded by forward stepwise likelihood ratio method

Then indexes with significance in univariate logistic analysis were then introduced into the multivariable logistic regression model using the forward stepwise (likelihood ratio) method (Table 2). The results indicated that T2DM duration (*P* = 0.014), TG (*P* = 0.006), SOD (*P* = 0.039), GSH-Px (*P* = 0.020), CRP (*P* = 0.047), TNF-α (*P* = 0.048), CSA (*P* = 0.010), SWV (*P* = 0.011), and NCV (*P* = 0.009) were independently risk factors for diabetic polyneuropathy. CAT, left CSA, and left NCV were excluded from the final model due to their lack of significant contribution to the model as determined by the forward stepwise selection process. Diabetic polyneuropathy risk was positively associated with T2DM duration (OR: 3.285, 95% CI, 1.276–8.453), TG (OR: 1.998, 95% CI, 1.224–3.262), CRP (OR: 1.641, 95% CI, 1.007–2.673), TNF-α (OR: 1.638, 95% CI. 1. 005-2.669), CSA (OR: 1.892, 95% CI, 1.161–3.082) and SWV (OR: 1.898, 95% CI, 1.160–3.107). SOD (OR: 0.592, 95% CI, 0.359–0.975), GSH-Px (OR: 0.555, 95% CI, 0.337–0.913), and NCV (OR: 0.522, 95% CI, 0.320–0.853) were negatively associated with the odds of diabetic polyneuropathy development.

### Correlation analysis between the VTIQ index and VAS score

We found that sciatic nerve width and **t**hickness do not correlate with the VAS score (*P* > 0.05). CSA on the left side of the sciatic nerve (*r* = 0.19, *P* = 0.027) and the right side (*r* = 0.28, *P* = 0.001) in patients with diabetic polyneuropathy had a low positive correlation with VAS score. SWV on the left side of the sciatic nerve (*r* = 0.22, *P* = 0.010) and the right side (*r* = 0.49, *P* < 0.001) showed a moderate positive correlation with the VAS score. VAS score showed a low negative correlation with NCV on the left side of the sciatic nerve (*r* = -0.25, *P* = 0.003) and the right side (*r* = -0.19, *P* = 0.031) (Table 3).

**Table 3:** Correlation analysis between VTIQ index and VAS score in diabetic polyneuropathy patients

Characteristics	*r*	*P*
**Width**		
Left	0.06	0.463
Right	0.01	0.943
**Thickness**		
Left	0.06	0.487
Right	0.06	0.473
**CSA**		
Left	0.19	0.027
Right	0.28	0.001
**SWV**		
Left	0.22	0.010
Right	0.49	0.000
**NCV**		
Left	-0.25	0.003
Right	-0.19	0.031

**Notes**: VTIQ, virtual touch tissue imaging quantification; VAS, visual analog scale; CSA, cross-sectional area; SWV, shear wave velocity; NCV, nerve conduction velocity. r, correlation coefficient

### Diagnostic efficacy of VTIQ-acquired indexes and NCV for diabetic polyneuropathy

**Fig. 3:**
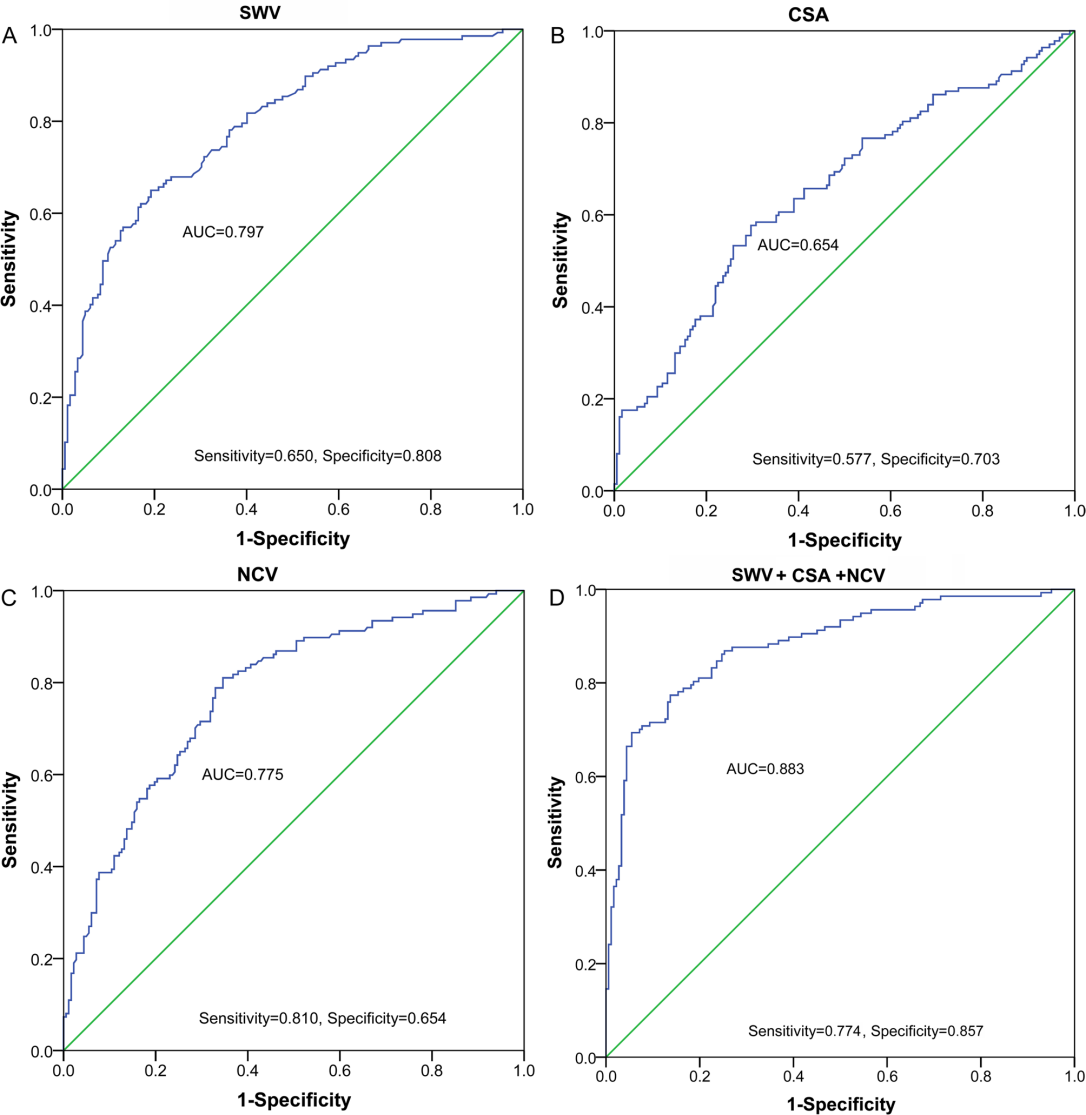
Diagnostic value analysis of CSA, SWV, NCV for diabetic polyneuropathy via plotting ROC curve. (**A**) Diagnostic value analysis of elastic modulus for diabetic polyneuropathy. (**B**) Diagnostic value analysis of CSA for diabetic polyneuropathy. C. Diagnostic value analysis of NCV for diabetic polyneuropathy. (**C**) Diagnostic value analysis of SWV, CSA, and NCV combination for diabetic polyneuropathy

The outcome of the ROC curve analysis showed that the Area Under the Curve (AUC) value of diabetic polyneuropathy judged by SWV was 0.797 (95% CI: 0.749–0.846, sensitivity: 0.650, specificity: 0.808, Table 4; Fig. 3A), cutoff value was 1.745 m/s, AUC value for CSA was 0.654 (95% CI: 0.593–0.715, sensitivity: 0.577, specificity: 0.703, Table 4; Fig. 3B), cutoff value is 70.79 mm^2^. The AUC for the NCV value was 0.775 (95% CI: 0.724–0.827, sensitivity: 0.810, specificity: 0.654, Table 4; Fig. 3C), with a critical value of 43.305 m/s. The AUC value for SWV + CSA was 0.823 (95% CI: 0.776–0.869, sensitivity: 0.781, specificity: 0.725, Table 4). In contrast, the AUC value for SWV + CSA + NCV was 0.883 (95% CI: 0.845–0.922, sensitivity: 0.774, specificity: 0.857, Table 4; Fig. 3D).

**Table 4:** Diagnostic value of features for diabetic polyneuropathy

Features	AUC (95%CI)	Sensitivity	Specificity	Cutoff value
SWV	0.797 (0.749–0.846)	0.650	0.808	1.745
CSA	0.654 (0.593–0.715)	0.577	0.703	70.79
SWV + CSA	0.823 (0.776–0.869)	0.781	0.725	0.392
NCV	0.775 (0.724–0.827)	0.810	0.654	43.305
SWV + CSA + NCV	0.883 (0.845–0.922)	0.774	0.857	0.488

**Notes**: CSA, cross-sectional area; NCV, nerve conduction velocity, AUC, area under curve; 95%CI, 95% confidence interval

## Discussion

The L4-S3 plexuses are connected to form the common peroneal and tibial nerves. These nerves leave the pelvis and are encircled by a general sheath of nerve fibers to form a single nerve trunk called the sciatic nerve, which is the largest branch of the sacral plexus, the largest nerve in the body, and is widely distributed in the lower limbs and manages sensation and movement in the lower limbs. There are many causes of sciatic neuropathy, such as Lumbar Herniated Disc (LHD), and DM [1, 31]. Currently, there are various ways to test the sciatic nerve, santos et al. found that the sciatic nerve had high hard properties presented by ultrasound elastography [32]. Andrade et al. found that sciatic nerve stiffness could be accurately assessed using the SWE technique [33]. The VTIQ technique quantifies nerve stiffness by measuring SWV at multiple points. Higher SWV values indicate faster propagation of the sound beam through the tissue, which also indicates greater stiffness and less elasticity of the tissue. In the present study, both the grey-scale B-mode ultrasound and VTIQ presented the structural and mechanical changes in the sciatic nerve of DPN patients. The increased CSA and SWV suggest that the nerve tissue has become more rigid and less compliant, which may contribute to the clinical symptoms of DPN, such as pain and sensory abnormalities, thus suggesting that the sciatic nerve elasticity was worse in patients with diabetic polyneuropathy, which is consistent with Chen’s study [34]. Our study used the VTIQ technique, which outperforms the traditional ultrasound imaging technique used by Chen et al. in precisely quantifying changes in nerve hardness and elasticity. It offers a more accurate indicator for early DPN diagnosis by multi-point measurement of SWV to reflect nerve elasticity. In addition, the VTIQ technique has been used to evaluate breast, thyroid, and prostate tumor diagnosis with greatly improved sensitivity, specificity, and accuracy [35]. It has been found that as the duration of diabetes increases, the peripheral nerve width, thickness, CSA, and SWV increase further, which is consistent with our outcome [34, 36]. Both studies confirm the importance of ultrasound in DPN diagnosis. However, unlike Chen et al.‘s focus on the tibial nerve, our study innovatively focused on the sciatic nerve. We found that its width, thickness, CSA, and SWV are significantly increased in DPN patients and are associated with clinical symptoms and nerve conduction function. These findings provide a more comprehensive perspective on the pathological changes in DPN across different nerves.

Increased sciatic nerve width, thickness, and CSA in patients with diabetic polyneuropathy, may be related to edema of the nerve bundle and [37]changes in nerve structure [38, 39]. In the case of long-term glucose metabolism disorder, it would lead to oxidative stress, mitochondrial dysfunction, inflammation-mediated and immune-mediated neurotoxicity, and the above pathways would progressively lead to the damage of Schwann cells, which provide energy and protection for neurons, and were crucial for the survival of peripheral nerves. At the same time, DPN led to microvascular lesions of the inner and outer nerve membranes, and the dysfunction of the endothelium, resulting in impaired nerve blood supply and hypoxia. The above pathophysiological changes ultimately lead to nerve demyelination, axonal conduction abnormality, and impaired neuronal regeneration, as well as increased nerve fibrosis and stiffness [40].

The ROC curves showed that SWV had higher diagnostic efficacy, but SWV + CSA + NCV resulted in a more favorable model diagnostic efficacy (Table 4), and thus SWV could be used as a reference index for diagnosing diabetic polyneuropathy. In addition, T2DM duration, TG, SOD, GSH-Px, CRP, and TNF-α, which were biochemical indicators, were associated with the odds of diabetic polyneuropathy development. CAT, left CSA, and left NCV were initially considered in the multivariable analysis but were excluded from the final model due to their lack of significant contribution to the model as determined by the forward stepwise (likelihood ratio) method. This method systematically evaluates the significance of each variable and retains only those that significantly improve the model fit. The exclusion of CAT, left CSA, and left NCV suggested that, while they may have shown significance in univariate analysis, it did not add meaningful predictive value in the presence of other variables in the multivariate model.

There are some limitations in this study, which need to be further improved. First, the sample size is small, and large samples or multi-center examination can reduce the error and increase the accuracy of the results. Sample size was determined based on the availability of eligible participants within the study period. No formal sample size calculation was performed prior to the study. This is acknowledged as a methodological limitation. But the statistical power was more than 0.9 indicating that our study had a high probability of detecting a true effect if one existed. Second, this study only studied the sciatic nerve, and it is necessary to further increase the number of peripheral nerves which are prone to changes to get more accurate data. Third, the correlation analysis revealed discrepancies in the correlation coefficients between the left and right sides of the sciatic nerve. While the exact cause of this difference is unclear, it may potentially be related to individual right/left dominance. Further research is needed to explore whether handedness or other factors contribute to these variations.

In conclusion, VTIQ is low cost, high accuracy, short time-consuming, non-invasive, and responsive technology, VITQ-acquired indexes are clinically important in the early diagnosis of peripheral neuropathy in patients with T2DM. Moreover, it can improve the diagnostic value of neurophysiological examination on sciatic neuropathy for T2DM patients. The findings are helpful for the early diagnosis and intervention for DPN.

## Supplementary Information

Below is the link to the electronic supplementary material.

Supplementary Material 1

Supplementary Material 2

## Data Availability

No datasets were generated or analysed during the current study.

## References

[1] Elafros MA, Andersen H, Bennett DL, Savelieff MG, Viswanathan V, Callaghan BC, et al. Towards prevention of diabetic peripheral neuropathy: clinical presentation, pathogenesis, and new treatments. Lancet Neurol. 2022;21(10):922–36. 36115364 10.1016/S1474-4422(22)00188-0 PMC10112836

[2] Iqbal Z, Azmi S, Yadav R, Ferdousi M, Kumar M, Cuthbertson DJ, et al. Diabetic peripheral neuropathy: epidemiology, diagnosis, and pharmacotherapy. Clin Ther. 2018;40(6):828–49. 29709457 10.1016/j.clinthera.2018.04.001

[3] Premkumar LS, Pabbidi RM. Diabetic peripheral neuropathy: role of reactive oxygen and nitrogen species. Cell Biochem Biophys. 2013;67(2):373–83. 23722999 10.1007/s12013-013-9609-5

[4] Sun J, Wang Y, Zhang X, Zhu S, He H. Prevalence of peripheral neuropathy in patients with diabetes: A systematic review and meta-analysis. Prim Care Diabetes. 2020;14(5):435–44. 31917119 10.1016/j.pcd.2019.12.005

[5] (CMA). EaCNGotNSotCMA, association ndgotnsotcm. Consensus on the diagnosis and treatment of diabetic peripheral neuropathy. Chin J Neurol. 2013;46(11).

[6] Mao W, Yip CW, Chen W. Complications of diabetes in china: health system and economic implications. BMC Public Health. 2019;19(1):269. 30841928 10.1186/s12889-019-6569-8 PMC6414024

[7] Pan C, Yang W, Jia W, Weng J, Tian H. Management of Chinese patients with type 2 diabetes, 1998–2006: the Diabcare-China surveys. Curr Med Res Opin. 2009;25(1):39–45. 19210137 10.1185/03007990802586079

[8] Liu Z, Fu C, Wang W, Xu B. Prevalence of chronic complications of type 2 diabetes mellitus in outpatients - a cross-sectional hospital based survey in urban China. Health Qual Life Outcomes. 2010;8:62. 20579389 10.1186/1477-7525-8-62 PMC2906445

[9] Yang D, Deng H, Luo G, Wu G, Lin S, Yuan L, et al. Demographic and clinical characteristics of patients with type 1 diabetes mellitus: A multicenter registry study in guangdong, China. J Diabetes. 2016;8(6):847–53. 26663759 10.1111/1753-0407.12366

[10] Guo K, Zhang L, Zhao F, Lu J, Pan P, Yu H, et al. Prevalence of chronic kidney disease and associated factors in Chinese individuals with type 2 diabetes: Cross-sectional study. J Diabetes Complicat. 2016;30(5):803–10. 10.1016/j.jdiacomp.2016.03.020 27068269

[11] Liu F, Bao Y, Hu R, Zhang X, Li H, Zhu D, et al. Screening and prevalence of peripheral neuropathy in type 2 diabetic outpatients: a randomized multicentre survey in 12 City hospitals of China. Diab/Metab Res Rev. 2010;26(6):481–9. 10.1002/dmrr.1107 20661939

[12] Kender Z, Jende JME, Kurz FT, Tsilingiris D, Schimpfle L, Sulaj A, et al. Sciatic nerve fractional anisotropy and neurofilament light chain protein are related to sensorimotor deficit of the upper and lower limbs in patients with type 2 diabetes. Front Endocrinol (Lausanne). 2023;14:1046690. 37008917 10.3389/fendo.2023.1046690 PMC10053786

[13] Selvarajah D, Kar D, Khunti K, Davies MJ, Scott AR, Walker J, et al. Diabetic peripheral neuropathy: advances in diagnosis and strategies for screening and early intervention. Lancet Diabetes Endocrinol. 2019;7(12):938–48. 31624024 10.1016/S2213-8587(19)30081-6

[14] Yang K, Wang Y, Li YW, Chen YG, Xing N, Lin HB, et al. Progress in the treatment of diabetic peripheral neuropathy. Biomed Pharmacother. 2022;148:112717. 35193039 10.1016/j.biopha.2022.112717

[15] Tang J, Shen H, Sun Y, Wang T, Li Y, Zhao Y. Research progress of high-frequency ultrasound in diagnosis of peripheral neuropathy. J Clin Ultrasound Med. 2019;21(12):930–3.

[16] Wang D, Xiang X, Yang Y. Research progresses of shear wave elastography for skin diseases Chinese. J Med Imaging Technol. 2023;39(05):781–4.

[17] Albers JW, Herman WH, Pop-Busui R, Martin CL, Cleary P, Waberski B. Subclinical neuropathy among diabetes control and complications trial participants without diagnosable neuropathy at trial completion: possible predictors of incident neuropathy? Diabetes Care. 2007;30(10):2613–8. 17644617 10.2337/dc07-0850 PMC2657957

[18] Bubnov RV. Ultrasonography diagnosis of peripheral neuropathy. The initial experience. Ultrasound Med Biol. 2011;37(8):S144–5.

[19] Bubnov R, Kalika L. EFFECTIVE RESTORING MOTION AND EFFECTIVE TREATMENT OF MYOFASCIAL AND NEUROPATHIC LOW BACK PAIN BY TARGATED DRY NEEDLING USING ULTRASOUND GUIDANCE. Ann Rheum Dis. 2019;78:1921–2.

[20] Bubnov RV. Evidence-based pain management: is the concept of integrative medicine applicable? EPMA J. 2012;3(1). 10.1186/1878-5085-3-13 PMC3533862 23088743

[21] Bubnov RV, Chaykovska I, Kotsyuba R. Ultrasound monitoring for chemotherapy complications in diabetic patients. Endocr Abstracts. 2025;54(110):EP378.

[22] Kelle B, Evran M, Ballı T, Yavuz F. Diabetic peripheral neuropathy: correlation between nerve cross-sectional area on ultrasound and clinical features. J Back Musculoskelet Rehabil. 2016;29(4):717–22. 26966822 10.3233/BMR-160676

[23] Gallardo E, Noto Y, Simon NG. Ultrasound in the diagnosis of peripheral neuropathy: structure Meets function in the neuromuscular clinic. J Neurol Neurosurg Psychiatry. 2015;86(10):1066–74. 25653385 10.1136/jnnp-2014-309599

[24] Røikjer J, Ejskjaer N. Diabetic peripheral neuropathy. Handb Exp Pharmacol. 2022;274:309–28. 35606621 10.1007/164_2022_585

[25] Dikici AS, Ustabasioglu FE, Delil S, Nalbantoglu M, Korkmaz B, Bakan S, et al. Evaluation of the tibial nerve with Shear-Wave elastography: A potential sonographic method for the diagnosis of diabetic peripheral neuropathy. Radiology. 2017;282(2):494–501. 27643671 10.1148/radiol.2016160135

[26] Üçeyler N, Schäfer KA, Mackenrodt D, Sommer C, Müllges W. High-Resolution ultrasonography of the superficial peroneal motor and Sural sensory nerves May be a Non-invasive approach to the diagnosis of vasculitic neuropathy. Front Neurol. 2016;7:48. 27064457 10.3389/fneur.2016.00048 PMC4812111

[27] Yu Y, Feng L. Objective methods for assessment of diabetic peripheral neuropathy. Int J Endocrinol METABOLISM. 2009;29(5):4.

[28] Umapathi T, Tan WL, Loke SC, Soon PC, Tavintharan S, Chan YH. Intraepidermal nerve fiber density as a marker of early diabetic neuropathy. Muscle Nerve. 2007;35(5):591–8. 17221881 10.1002/mus.20732

[29] Zhang C, Li M, Jiang J, Zhou Q, Xiang L, Huang Y, et al. Diagnostic value of virtual touch tissue imaging quantification for evaluating median nerve stiffness in carpal tunnel syndrome. J Ultrasound Med. 2017;36(9):1783–91. 28436592 10.1002/jum.14213

[30] Lai ZH, Yang SP, Shen HL, Luo Y, Cai XH, Jiang WT, et al. Combination of high-frequency ultrasound and virtual touch tissue imaging and quantification improve the diagnostic efficiency for mild carpal tunnel syndrome. BMC Musculoskelet Disord. 2021;22(1):112. 33499842 10.1186/s12891-021-03982-7 PMC7836488

[31] Kara M, Özçakar L, Tiftik T, Kaymak B, Özel S, Akkuş S, et al. Sonographic evaluation of sciatic nerves in patients with unilateral sciatica. Arch Phys Med Rehabil. 2012;93(9):1598–602. 22453115 10.1016/j.apmr.2012.03.013

[32] Santos R, Armada P. Biology. Sciatic nerve hardness measurement by using ultrasound elastography. Ultrasound Med. 2013;39.

[33] Andrade RJ, Nordez A, Hug F, Ates F, Coppieters MW, Pezarat-Correia P, et al. Non-invasive assessment of sciatic nerve stiffness during human ankle motion using ultrasound shear wave elastography. J Biomech. 2016;49(3):326–31. 26725218 10.1016/j.jbiomech.2015.12.017

[34] Chen X. A multimodal ultrasound study of the tibial nerve in patients with type 2 diabetic peripheral neuropathy [硕士]2023.

[35] Zhao X, Zhai H, Zhang Q, Zhao Q, Sun N, Wang B. Analysis of the accuracy of quantitative acoustic palpation tissue imaging techniques for diagnosing benign and malignant breast tumours and the factors influencing them. Chongqing Med Sci. 2016;45(21):2989–91.

[36] Singh KP, Gupta K, Kataria N, Arora V, Nagpal N. High-resolution ultrasonography of the Sural nerve in diabetic peripheral neuropathy. J Ultrasonography. 2020;20(81):e83–9. 10.15557/JoU.2020.0013 PMC7409546 32609965

[37] Li J. Clinical analysis of diabetes mellitus combined with chronic inflammatory demyelinating polyradiculoneuropathy. New World Diabetes. 2019;22(12):29–30.

[38] Ozhigrad., Zhang L, Yu Y. Advances in the relationship between ion channels and diabetic peripheral neuralgia. Chin J Neurosurg. 2018;34(3):321–4.

[39] Yang X, Li M. Clinical value of high-frequency ultrasound and elastography in the assessment of peripheral neuropathy in type 2 diabetes mellitus. J Practical Med. 2020;36(03):390–4.

[40] Sloan G, Selvarajah D, Tesfaye S. Pathogenesis, diagnosis and clinical management of diabetic sensorimotor peripheral neuropathy. Nat Reviews Endocrinol. 2021;17(7):400–20. 10.1038/s41574-021-00496-z 34050323

