# Supplementary material for: Quantitative study and related factors analysis of sciatic neuropathy in type 2 diabetes mellitus patients by elastic imaging virtual tissue imaging quantification technique
Source: Hereditas. 2025 Oct 10;162:207. doi: 10.1186/s41065-025-00565-7 (PMC12512427; doi:10.1186/s41065-025-00565-7)
Supplement: Supplementary file 1 — Supplementary Material 1 [file 41065_2025_565_MOESM1_ESM.docx]

**Supplementary Table 1**. Difference of VTIQ index between different sides.

| Index | Control (n=198) | | | T2DM (n=182) | | | DPN (n=137) | | |
| --- | --- | --- | --- | --- | --- | --- | --- | --- | --- |
|  | Left | Right | *P* | Left | Right | *P* | Left | Right | *P* |
| Width (mm) | 13.25±1.26 | 13.42±1.28 | 0.182 | 13.99±2.01 | 13.96±1.98 | 0.863 | 14.70±2.10 | 14.81±1.99 | 0.657 |
| Thickness (mm) | 5.16±0.62 | 5.20±0.68 | 0.493 | 5.76±0.87 | 5.92±0.94 | 0.084 | 6.86±1.19 | 7.02±1.25 | 0.277 |
| CSA | 54.67±13.63 | 55.54±12.99 | 0.516 | 64.03±14.19 | 64.36±13.36 | 0.816 | 70.61±13.42 | 72.00±14.78 | 0.418 |
| SWV | 1.25±0.21 | 1.27±0.22 | 0.221 | 1.55±0.24 | 1.55±0.24 | 0.984 | 1.80±0.23 | 1.83±0.25 | 0.218 |

**Notes**: T2DM, type 2 diabetes mellitus; DPN, diabetic polyneuropathy; CSA, cross-sectional area; SWV, shear wave velocity.
