# Supplementary figures and images for: Quantitative study and related factors analysis of sciatic neuropathy in type 2 diabetes mellitus patients by elastic imaging virtual tissue imaging quantification technique
Source: Hereditas. 2025 Oct 10;162:207. doi: 10.1186/s41065-025-00565-7 (PMC12512427; doi:10.1186/s41065-025-00565-7)

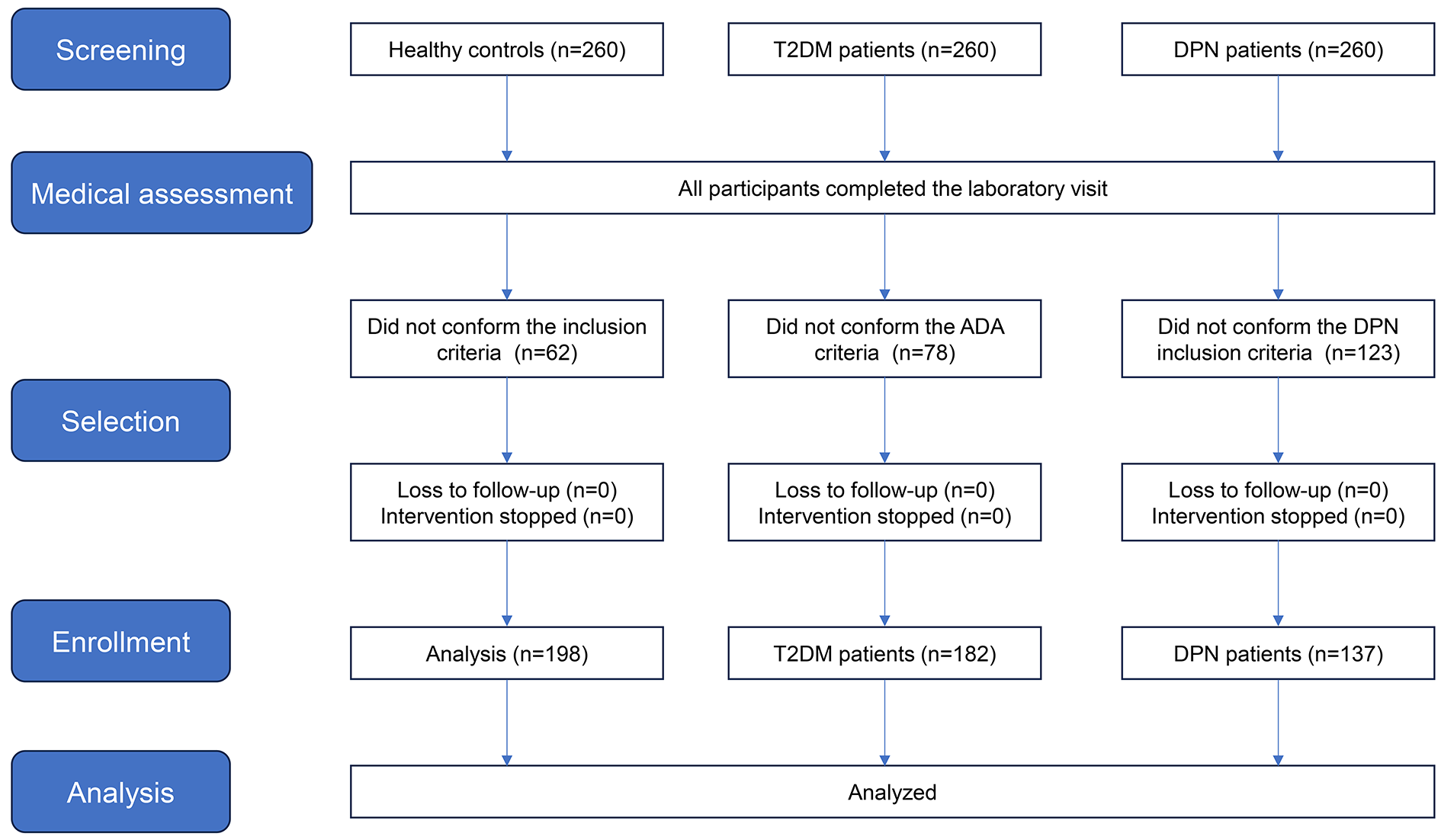

Supplement: Supplementary file 2 — Supplementary Material 2 [file 41065_2025_565_MOESM2_ESM.tif]
